# Exploring Variations in Lumbar Canal Width: An MRI Study on Asymptomatic Patients by Age and Gender

**DOI:** 10.3390/jcm13226775

**Published:** 2024-11-11

**Authors:** Betül Tiryaki Baştuğ

**Affiliations:** Radiology Department, Bilecik Şeyh Edebali University Medical Faculty, 11000 Bilecik, Türkiye; betultryak@yahoo.com

**Keywords:** lumbar spinal stenosis, MRI, spinal canal narrowing, age-related stenosis, gender differences, L4-5 stenosis, L5-S1 stenosis, asymptomatic patients

## Abstract

**Background:** Lumbar spinal stenosis is a common degenerative condition, especially in older adults, leading to significant morbidity. Age, gender, and lumbar level variations in spinal canal width are critical in assessing stenosis risk. Many patients exhibit radiographic narrowing without clinical symptoms. This study analyzed the risk of lumbar stenosis by age, gender, and lumbar levels (L1-S1) using the MRI of patients without clinical signs of narrowing. **Materials and Methods:** This retrospective study included 120 patients, aged 20 to 75, who underwent lumbar MRI for unrelated reasons. Spinal canal widths were measured at five lumbar levels (L1-2 to L5-S1), and stenosis risk was classified as low, borderline, or high based on narrowing thresholds. Data were grouped by age and gender to assess the stenosis risk distribution. **Results:** The analysis revealed a progressive increase in stenosis risk from the upper to lower lumbar levels. At L4-5 and L5-S1, females aged 61–75 exhibited the highest proportions in borderline- and high-risk categories. While most patients were classified as low risk, many older patients showed radiographic narrowing without clinical symptoms. Males generally had wider canals and lower risk. **Conclusions:** Age and gender significantly impact lumbar stenosis risk, particularly in older females. The findings highlight the importance of clinical correlation despite radiographic evidence of stenosis and suggest potential for AI-based detection systems in enhancing assessments of spinal canal narrowing.

## 1. Introduction

Understanding the normal anatomy and variations of the lumbar spinal canal is essential for the accurate diagnosis and effective treatment of lumbar spine conditions. The lumbar spine is critical for maintaining structural integrity and facilitating movement in the human body. The spinal canal, which houses the cauda equina and nerve roots, plays a vital role in the motor and sensory functions of the lower limbs [1,2]. Changes in the anatomy of the lumbar spine, particularly in the width of the spinal canal, can lead to conditions such as lumbar spinal stenosis. This condition is characterized by the narrowing of the spinal canal, which may result in neural compression and significant clinical symptoms, including lower back pain and neurogenic claudication [3,4].

Understanding the dimensions of the spinal canal is essential for the accurate diagnosis and effective treatment of lumbar spine conditions. The dimensions are influenced by various factors, including age, gender, and the degenerative changes associated with aging [5,6,7]. While it is well established that the spinal canal narrows with age due to degenerative processes such as disc bulging, ligament hypertrophy, and osteophyte formation, the precise influence of aging and gender on spinal canal dimensions remains an area of active research [8]. Differentiating between normal anatomical variations and pathological narrowing is crucial for clinical intervention.

The aging spine undergoes anatomical changes that can lead to spinal stenosis, including disc degeneration, facet joint hypertrophy, and thickening of the ligamentum flavum [9,10]. Although spinal stenosis is common in older adults, not all narrowing of the spinal canal is pathological; thus, establishing normative data on spinal canal dimensions is important to distinguish between age-related normal variations and clinically significant stenosis.

Furthermore, gender differences in spinal canal dimensions have been observed, with studies suggesting that males tend to have wider spinal canals than females [11,12]. This disparity has implications for the diagnosis and management of spinal conditions as females may be at a higher risk for developing symptomatic stenosis due to relatively narrower canal dimensions [13]. However, the interaction between age, gender, and spinal canal dimensions is complex and warrants further investigation.

Magnetic resonance imaging (MRI) is the gold standard for evaluating the lumbar spinal canal due to its high-resolution imaging capabilities, which allow for detailed visualization of soft-tissue structures without the use of ionizing radiation [14]. This modality enables precise measurements of spinal canal dimensions at various lumbar levels, which is critical for diagnosing conditions such as lumbar spinal stenosis. Despite its extensive use, there remains a lack of normative data on spinal canal dimensions across different age groups and between sexes, particularly in asymptomatic individuals. Most existing studies have concentrated on pathological populations, making it challenging to define what constitutes a “normal” range for spinal canal widths [15].

By excluding patients with known lumbar spine conditions or symptoms, this study seeks to provide a clearer understanding of how spinal canal width varies naturally across the population. Our primary objective is to assess lumbar spinal canal width at five vertebral levels (L1-2, L2-3, L3-4, L4-5, and L5-S1) in individuals aged 20 to 75 years. We will analyze the relationship between spinal canal width, age, and gender to establish normative data that can serve as a reference for clinicians in the diagnosis and management of spinal conditions.

Focusing on asymptomatic individuals will help identify baseline anatomical variations in spinal canal width that may assist in the early detection of abnormal narrowing. This is particularly important in the context of aging, where normal degenerative changes may mimic pathological conditions, leading to overdiagnosis and overtreatment. Understanding how spinal canal width differs between males and females could facilitate more personalized approaches in the diagnosis and treatment of lumbar spine conditions.

The results of this study will not only contribute to the understanding of normal spinal canal anatomy, but may also provide valuable insights into the natural history of spinal stenosis and other degenerative lumbar conditions. Establishing a clear baseline of spinal canal dimensions across different age groups and sexes will aid in the development of better diagnostic criteria and treatment guidelines for conditions such as lumbar spinal stenosis, disc herniation, and other spinal pathologies.

In conclusion, assessing lumbar spinal canal dimensions is crucial for the accurate diagnosis and management of lumbar spine conditions. By providing normative data on spinal canal width across a diverse cohort, this study seeks to bridge the current knowledge gap and offer clinicians a robust reference for differentiating between normal anatomical variations and pathological changes. The findings will enhance clinical decision making and may have implications for the prevention and early intervention of spinal disorders, ultimately improving patient outcomes and quality of life.

These purposes clearly articulate the primary objectives of our research:

✓ To measure the lumbar spinal canal widths in asymptomatic individuals across different age groups (20–75 years) and genders.
✓ To analyze the variations in canal dimensions and their potential implications for clinical practice, particularly concerning the early identification of individuals at risk for developing symptomatic lumbar spinal stenosis.
✓ To contribute normative data that can aid clinicians in differentiating between normal anatomical variations and pathological conditions.

## 2. Materials and Methods

### 2.1. Study Design

This study is a retrospective analysis aimed at evaluating the lumbar spinal canal width in individuals across different age groups and genders. This study was conducted using lumbar magnetic resonance imaging (MRI) data from 120 randomly selected patients who underwent MRI for reasons unrelated to clinical signs of canal narrowing between January 2023 and January 2024. These individuals were selected without regard to their clinical condition as the primary objective was to measure anatomical dimensions rather than diagnose pathology. The data collected included measurements of the spinal canal width at five specific lumbar levels (L1-2, L2-3, L3-4, L4-5, and L5-S1), along with each patient’s age and gender.

### 2.2. Ethical Considerations

This study was conducted in accordance with the guidelines detailed in the Declaration of Helsinki, and it was approved by the Ethics Committee of Non-Interventional Clinical Research of Bilecik Seyh Edebali University (protocol code: Decision No. 5 of the 4th meeting, and date of approval was 27 March 2024). Informed consent was obtained from all of the subjects involved in this study.

### 2.3. Patient Selection and Data Collection

A total of 120 patients were randomly selected from those who had undergone lumbar MRI scans for reasons unrelated to clinical signs of canal narrowing. The most common reasons for MRI scans included evaluations for trauma, assessments related to other non-lumbar medical conditions, and general health screenings.

It is important to note that these patients were not specifically referred for MRI as part of this study; instead, we utilized data from individuals who underwent MRI for legitimate clinical reasons. This approach was taken to minimize bias and ensure that the anatomical measurements were not influenced by pre-existing lumbar pathologies.

Due to the potential overlap with the clinical presentation of lumbar spinal stenosis, most of the patients fell within the exclusion criteria. The majority of the patients we reported consisted of trauma patients, individuals suspected of having musculoskeletal disorders such as fibromyalgia, inflammatory, or rheumatological diseases like rheumatoid arthritis, ankylosing spondylitis, and spondyloarthritis, as well as malignancies, including metastatic diseases and infectious processes like tuberculosis osteomyelitis.

We did not include specific clinical data, such as comorbidities or diagnoses, in this study because our primary objective was to evaluate anatomical dimensions in a normative context.

### 2.4. Inclusion Criteria

- Patients aged 20 to 75 years; this age range was chosen to capture a broad spectrum of anatomical variations associated with aging, as well as to facilitate comparisons across different age groups.
- Patients who underwent lumbar MRI for non-lumbar-related complaints or general health assessments. This criterion was essential to minimize bias and ensure that the imaging was not influenced by prior or existing lumbar pathologies that could affect spinal canal dimensions.

High-quality imaging at all five lumbar levels (L1-2, L2-3, L3-4, L4-5, and L5-S1) was conducted to ensure accurate measurements of the spinal canal width. High-resolution scans were necessary to ensure that clear and precise measurements could be obtained for the analysis.

### 2.5. Exclusion Criteria

- Any previous history of lumbar spine surgery. This was crucial as surgical interventions can significantly alter the anatomical structure of the spine and skew the results.
- Patients with severe spinal deformities or conditions affecting spinal anatomy, such as congenital anomalies or severe scoliosis, were also excluded. These conditions could introduce variability in the measurements and compromise the integrity of the study.
- Incomplete or low-quality MRI scans where clear measurements could not be obtained. This step ensured that the data collected were reliable and valid for the research purposes.

### 2.6. Imaging and Measurement Protocol

All MRI scans were performed using a 1.5 Tesla MRI scanner, with the imaging protocol including both T1- and T2-weighted sequences in the axial and sagittal planes. These images provided clear visualization of the spinal canal at each lumbar level. The spinal canal width was measured at the widest point of the canal at five lumbar levels (L1-2, L2-3, L3-4, L4-5, and L5-S1) using the axial plane. A single experienced radiologist, with 22 years of experience in MRI interpretation, manually performed all of the measurements. Measurements were taken in millimeters (mm), ensuring consistency.

#### MRI Acquisition Details

The MRI examinations were performed using a Siemens Magnetom Essenza 1.5 Tesla MRI scanner, which produces high-resolution images with an enhanced signal-to-noise ratio and spatial resolution. Prior to the MRI scan, patients were instructed to remove any metallic objects, including jewelry and accessories, to avoid interference with the magnetic field. They were positioned supine on the MRI table, with appropriate cushions used to ensure comfort and minimize movement during the scan. A combination of T1-weighted and T2-weighted sequences was utilized to provide comprehensive views of the lumbar spine. T1-weighted imaging, employed for assessing the anatomy of the vertebrae and surrounding soft tissues, was typically acquired with the following parameters: a TR (repetition time) of 400 ms, a TE (echo time) of 10 ms, a slice thickness of 4 mm, and a field of view (FOV) of 300 mm. For this study, the spatial resolution typically ranged from 0.5 mm to 1.0 mm depending on the matrix size used during imaging. The T2-weighted imaging, used for evaluating disc pathology and spinal canal dimensions, was typically acquired with parameters of a TR of 3000 ms, a TE of 90 ms, a slice thickness of 4 mm, and a FOV of 300 mm. Localizer images were obtained in the axial, sagittal, and coronal planes to accurately position the slices at the lumbar levels (L1-S1) of interest. Axial slices were then obtained at each lumbar level (L1-2, L2-3, L3-4, L4-5, and L5-S1) to measure the spinal canal width, with the number of slices and their orientation adjusted to ensure comprehensive coverage of the lumbar region. The total acquisition time for each MRI examination typically ranged from 20 to 30 min, depending on the specific protocol and sequences used. The acquired images were reconstructed and processed using dedicated software to enhance the visualization and measurement of spinal canal dimensions. Image quality was assessed to ensure clarity and diagnostic value before analysis. Finally, a radiologist with 23 years of experience evaluated the images for any artifacts or abnormalities that could affect the analysis.

### 2.7. Data Organization

The dataset consisted of age, gender, and spinal canal width measurements at five lumbar levels for each patient. The age range of the patients was divided into three groups, i.e., 20–40 years, 41–60 years, and 61–75 years, to evaluate how the canal width changes across different stages of life. Gender-specific analysis was also conducted to investigate the potential differences in lumbar spinal canal dimensions between males and females.

#### 2.7.1. Rationale for Measuring the Widest Part of the Canal

The rationale for measuring the widest part of the spinal canal lies in its ability to provide essential insights into canal anatomy. The widest part often reflects the overall anatomical structure, allowing for a better evaluation of individual variations. Serving as a standardized reference point across different patients and populations, this measurement facilitates comparisons that are crucial for identifying abnormal canal dimensions and informing clinical decisions regarding interventions. Additionally, the correlation between the canal width and the presence of symptoms can aid clinicians in predicting which patients may develop symptomatic stenosis in the future, thus offering vital predictive value for proactive management and treatment planning. By measuring this widest section, healthcare providers can also determine the need for further diagnostic evaluation. In research contexts, quantifying the widest part of the canal helps establish normative data, which is critical for understanding how various factors—such as age and gender—impact spinal canal dimensions, ultimately contributing to the development of improved diagnostic criteria and treatment protocols.

#### 2.7.2. Measurement Technique

Canal Diameter: Measurements are often taken at the narrowest point of the canal anteroposterior (AP), and thresholds of < 10 mm in the AP diameter may indicate significant narrowing.

#### 2.7.3. Measurement Methodology

1. Image Acquisition:
  - MRI scans of the lumbar spine were obtained using a Siemens Magnetom Essenza 1.5 Tesla. The scans included high-resolution axial and sagittal images that provided clear visualization of the spinal canal.
2. Measurement Procedure:
  - The spinal canal width was measured at five lumbar levels: L1-2, L2-3, L3-4, L4-5, and L5-S1.
  - Measurements were taken at the widest part of the canal to ensure consistency and accuracy.
  - For each level, the following procedures were followed:
    ✓ Selection of Axial Images: Axial images at each lumbar level were identified using the MRI protocol.
    ✓ Drawing Contours: A region of interest (ROI) was manually drawn around the spinal canal on the selected axial images. This contouring delineates the canal boundaries for accurate area and diameter measurements.
    ✓ Measurement Parameters: The anteroposterior (AP) diameters of the canal were measured along with the cross-sectional area.
3. Software Utilized:
  - Measurements were conducted using RadiAnt DICOM Viewer (2024.1). This software allows for precise measurements of anatomical structures and provides tools for analyzing MRI data effectively.
  - The software features include the following:
    ✓ Interactive Measurement Tools: To accurately measure distances and areas, ensuring reliable data collection.
    ✓ 3D Reconstruction: If applicable, to visualize the spinal canal in three dimensions for better understanding and assessment.
    ✓ Data Export Options: To export measurement data for further statistical analysis.
4. Quality Control:
  - All measurements were performed by a trained radiologist with 23 years of experience.

### 2.8. Statistical Analysis

The data were processed and analyzed using SPSS version 23.0 software. Descriptive statistics, including means and standard deviations, were calculated for each lumbar level and for each age and gender group. Independent *t*-tests were used to compare the spinal canal widths between male and female patients. One-way analysis of variance (ANOVA) was employed to assess the variations in spinal canal width across different age groups. Post hoc tests were applied to identify specific differences between age groups when significant results were found. A *p*-value of less than 0.05 was considered statistically significant in all analyses. Additionally, linear regression analysis was performed to examine the correlation between the age and spinal canal width at each lumbar level.

### 2.9. Outcome Measures

The primary outcome of interest was the variation in lumbar spinal canal width across different age groups and between genders. Secondary outcomes included the identification of trends in canal width reduction or expansion with aging and whether these trends differed between men and women. The results are intended to contribute to the understanding of normal anatomical variation in lumbar spinal canal dimensions, potentially aiding in future clinical assessments and treatment planning for spinal conditions.

## 3. Results

The analysis of lumbar spinal canal width data yielded several notable findings regarding variations across age groups and between genders. The study population, comprising 120 individuals aged between 20 and 75, was divided into three age groups: 20–40 years, 41–60 years, and 61–75 years. Measurements were taken at five lumbar levels (L1-2, L2-3, L3-4, L4-5, and L5-S1) for each participant, and the data were analyzed for age-related and gender-based differences in spinal canal width.

### 3.1. Spinal Canal Width by Age Group

The results indicate that lumbar spinal canal width generally decreases with age across all lumbar levels. This trend was most pronounced in the 61–75 age group, where the average spinal canal width was smallest at each measured lumbar level compared to the younger age groups.

- Age Group 20–40: In the youngest age group, the average spinal canal width was relatively wide across all lumbar levels, with the L1-2 level having an average width of 19.3 mm and the L5-S1 level measuring 18.5 mm. This group exhibited the largest spinal canal widths across all levels, reflecting minimal degenerative changes at these ages.
- Age Group 41–60: The intermediate age group showed a slight decrease in spinal canal width compared to the 20–40 age group. The average width at the L1-2 level was 19.1 mm, and it was 17.9 mm at the L5-S1 level. This reduction is indicative of early age-related degenerative changes, though the decrease in width is not yet substantial.
- Age Group 61–75: In the oldest age group, the average spinal canal width narrowed considerably, particularly at the L3-4 and L4-5 levels. At the L1-2 level, the average width was 18.5 mm, and it was 16.2 mm at L5-S1, reflecting significant narrowing compared to the younger groups. This finding aligns with the known degenerative changes that occur with aging, such as disc degeneration and ligamentous hypertrophy, which contribute to a reduced spinal canal diameter.

The differences in spinal canal width between the age groups were statistically significant (*p* < 0.05), particularly at the L3-4 and L4-5 levels, where age-related narrowing was most prominent (Table 1).

### 3.2. Spinal Canal Width by Gender

When analyzing gender differences, it was observed that males generally had wider spinal canals than females at most lumbar levels, though the differences were modest.

- Females: The average spinal canal width for females across all lumbar levels ranged from 16.1 mm at the L5-S1 level to 18.6 mm at the L1-2 level. The narrowest point was consistently at the L5-S1 level, which is consistent with previous research indicating that the distal lumbar levels tend to show greater narrowing, especially in older adults. The relatively narrower canal in females may be a contributing factor to the higher prevalence of lumbar stenosis symptoms reported in older women.
- Males: The average spinal canal width for males ranged from 17.5 mm at the L5-S1 level to 18.6 mm at the L1-2 level. Males exhibited slightly wider spinal canals than females across all lumbar levels. The most significant difference was observed at the L4-5 level, where males had an average canal width of 17.7 mm compared to 16.9 mm in females. Although the difference in spinal canal width between genders was not statistically significant at every lumbar level, it suggests a tendency for males to have a wider canal, which may be a protective factor against developing spinal stenosis (Table 2).

### 3.3. Age and Gender Interactions

The interaction between age and gender revealed some interesting trends. In the younger age group (20–40 years), males had substantially wider spinal canals compared to females, particularly at the L1-2 and L2-3 levels. However, this difference between genders appeared to decrease with age. By the time individuals reached the 61–75 age group, the narrowing of the spinal canal due to age-related degenerative changes appeared to affect both genders similarly, with no significant gender differences in canal width at most lumbar levels.

The narrowing of the spinal canal with age, particularly at the L3-4 and L4-5 levels, was more pronounced in females than in males. This finding may partly explain why females tend to experience symptoms of lumbar spinal stenosis at an earlier age than males as they start with narrower canal dimensions and undergo similar age-related narrowing (Table 3).

### 3.4. Statistical Analysis and Significance

Statistical analysis using independent *t*-tests and ANOVA revealed that the differences in spinal canal widths between the age groups were statistically significant, particularly at the L3-4 and L4-5 levels (*p* < 0.05). The post hoc tests confirmed that the most significant reductions in canal width occurred between the 41–60 and 61–75 age groups, indicating that a late stage of life is associated with a notable decrease in lumbar spinal canal dimensions.

Gender-based differences, while present, did not reach statistical significance (*p* > 0.05) at every lumbar level, although males generally exhibited wider spinal canals than females across most levels. The exception was at the L4-5 and L5-S1 levels in the oldest age group, where females showed greater narrowing compared to males (Table 4).

Table 5 summarizes the percentage differences in lumbar spinal canal widths between males and females across three age groups (20–40, 41–60, and 61–75) at specific lumbar levels (L1-2 and L4-5). The data reveal that males generally have wider spinal canals compared to females, with the most pronounced differences observed in the younger age groups. For instance, in the 20–40 age group, the males exhibited a 14.9% wider canal at the L1-2 level and a 14.1% difference at the L4-5 level. Statistical analyses performed using independent *t*-tests to compare the means between genders for each age group indicated significant differences, with *p*-values being less than 0.05. As the patients’ age increased, particularly in the 61–75 age group, the differences between genders narrowed significantly. In this group, the percentage difference at the L1-2 level was negligible (−0.6%), while, at the L4-5 level, there was a small but positive difference (2.7%). However, the statistical significance for these older patients suggests that the anatomical narrowing of the spinal canal may reduce gender disparities, as indicated by *p*-values greater than 0.05. Overall, these findings highlight the importance of considering age and gender when assessing lumbar spinal canal anatomy, particularly in clinical settings where stenosis risk may be a concern.

Table 6 presents the correlation coefficients between the age of patients and the cross-sectional area of the lumbar spinal canal at various lumbar levels (L1-2, L2-3, L3-4, L4-5, and L5-S1). The analysis revealed a consistent negative correlation across all levels, indicating that, as age increases, the cross-sectional area of the spinal canal tends to decrease. For instance, at the L4-5 level, the correlation coefficient was −0.5, suggesting a moderate negative relationship, and it was statistically significant with a *p*-value of less than 0.05. This finding reinforces the notion that age-related degenerative changes contribute to spinal canal narrowing, and it was particularly evident in older populations. Similarly, the L5-S1 level also showed a significant negative correlation (−0.4), emphasizing that this region is particularly vulnerable to changes associated with aging. The significant *p*-values across all levels underscore the importance of considering age as a critical factor in assessing lumbar spinal canal dimensions. These results provide valuable insights into the anatomical changes that occur with aging and highlight the potential clinical implications for the diagnosis and management of conditions such as lumbar spinal stenosis.

### 3.5. Summary of Key Findings

1. Age-related narrowing: The lumbar spinal canal tends to narrow with increasing age, particularly at the L3-4 and L4-5 levels, with the 61–75 age group showing the most significant reduction in spinal canal width.
2. Gender differences: Males generally have wider spinal canals than females, but this difference is most pronounced in younger age groups. By the age of 61–75, the effect of age-related narrowing reduces the gender differences.
3. Clinical implications: These findings suggest that both age and gender should be considered when evaluating patients for lumbar spinal stenosis. Females may be at a higher risk for earlier onset of symptoms due to narrower baseline canal widths and more pronounced narrowing with age.

In the analysis of lumbar stenosis risk based on spinal canal widths, our cohort showed a predominantly low risk for stenosis across all age groups and genders. Notably, no patients were classified as being at high risk for stenosis, and only a small subset fell into the borderline category. Specifically, in the 41–60 and 61–75 age groups, a few individuals demonstrated borderline canal widths at the L3-4, L4-5, and L5-S1 levels, particularly among females. However, the majority of patients across all age groups maintained canal widths within the low-risk range. Importantly, none of the patients in our study exhibited the clinical symptoms typically associated with lumbar spinal stenosis, further reinforcing the finding that reduced spinal canal width in this cohort did not correspond to symptomatic stenosis (Table 7 and Table 8).

### 3.6. Lumbar Stenosis Risk by Level

The analysis of stenosis risk across the five lumbar levels (L1-2, L2-3, L3-4, L4-5, and L5-S1) revealed significant variations in the proportion of patients at risk for lumbar spinal stenosis, with a clear trend of increased risk at lower lumbar levels. The majority of patients, across all levels, fell into the low-risk category, indicating that stenosis is not a common finding in this cohort. However, the distribution of borderline- and high-risk cases shows that the risk of stenosis increased progressively as we moved from the upper lumbar levels (L1-2) to the lower levels (L5-S1), which are more prone to degenerative changes.

### 3.7. L1-2 and L2-3 Levels

The L1-2 and L2-3 levels exhibited the lowest stenosis risk, with the majority of patients classified as low risk across the respective age groups. The 41–60 age group showed a borderline risk at the L3-4 level, while the 61–75 age group presented a borderline risk at the L5-S1 level. The presence of high-risk patients was nearly absent at these upper levels. These findings are consistent with the expected anatomical configuration of the upper lumbar spine, where the canal tends to be wider and less affected by degenerative changes compared to lower levels. The minimal occurrence of high-risk cases at L1-2 and L2-3 suggests that stenosis rarely develops here in the absence of more advanced degenerative disease.

### 3.8. L3-4 Level

At the L3-4 level, the proportion of patients in the low-risk category decreased to 82.9%, while the percentage of patients at borderline risk rose to 14.6%. This lumbar level is a transitional zone between the upper and lower lumbar spine, and it is often where the first signs of degenerative narrowing begin to appear. The small but notable increase in high-risk cases (2.4%) at this level suggests that some individuals may begin to experience structural changes leading to stenosis as they age.

### 3.9. L4-5 Level

The L4-5 level demonstrated a further increase in stenosis risk, with only 78.1% of patients classified as low risk. The proportion of borderline-risk patients rose to 14.6%, and the high-risk category climbed to 7.3%, marking this level as a more vulnerable site for stenosis development. This is in line with the known biomechanical stresses at the L4-5 level, which is subject to significant load-bearing and motion, predisposing it to degenerative changes such as disc herniation and ligamentum flavum hypertrophy. This level is commonly affected in clinical cases of lumbar stenosis, and the increasing high-risk proportion reflects the potential for symptomatic stenosis.

### 3.10. L5-S1 Level

The L5-S1 level showed the highest risk of stenosis in this cohort. While 73.2% of patients remained in the low-risk category, the percentage of borderline-risk patients increased to 14.6%, and 12.2% of patients were classified as high risk. This lumbar level is known for bearing the greatest mechanical stress due to its position at the base of the spine, often leading to degenerative changes such as disc collapse and facet joint osteoarthritis. The relatively higher proportion of high-risk cases at L5-S1 indicates that this level is the most likely site for stenosis to develop, even in asymptomatic individuals. These findings suggest that a close monitoring of this level is warranted in patients at risk for lumbar stenosis, particularly as they age.

### 3.11. Overall Insights

The stenosis risk increased progressively from the L1-2 to L5-S1 levels, with the lowest risk observed in the upper lumbar spine and the highest risk in the lower lumbar spine. This pattern is consistent with the natural history of spinal degeneration, where the lower lumbar levels experienced greater mechanical wear and tear. The presence of borderline-risk cases across all levels, but especially at the L4-5 and L5-S1 levels, underscores the importance of regular imaging and monitoring to prevent the progression of subclinical stenosis into symptomatic disease.

Importantly, it is worth noting that despite the presence of borderline- and high-risk cases, none of the patients in this study exhibited clinical symptoms of stenosis. This finding reinforces the notion that stenosis is a gradual process, and even individuals with radiographic evidence of narrowing may remain asymptomatic for extended periods. Thus, while radiographic stenosis is an important finding, clinical correlation remains essential in determining the need for intervention (Table 9).

When we take a closer look at the L4-5 level, where the stenosis risk appeared to be more pronounced, the stenosis risk showed variation across age groups and genders. Among patients aged 20–40, the majority were in the low risk category, with only one female patient classified as borderline. For patients aged 41–60, the females exhibited a higher proportion of risk, with one patient falling into the high risk category. Males in this age group showed more favorable results, with only one patient in the borderline-risk category. In the 61–75 age group, females again demonstrated a higher proportion of borderline-risk cases, with two patients in this category and the remainder in the low risk group. Overall, females, particularly in the older age groups, appeared to have a higher risk of stenosis at the L4-5 level compared to males. The increased risk at the L4-5 level could be attributed to the significant mechanical stress and degenerative changes this region endures, such as disc herniation and ligamentum flavum hypertrophy. This level often serves as a transition point between the more stable upper lumbar levels and the highly mobile L5-S1, making it particularly vulnerable to narrowing. Among our study population, females aged 61–75 showed the highest risk of stenosis at the L4-5 level, reflecting the age-related degenerative changes that were more pronounced in this group. Males, on the other hand, generally exhibited lower risk across all age groups, further supporting the gender disparity in spinal canal narrowing at this level (Table 10).

When we took a closer look at the L5-S1 level, where the stenosis risk was particularly high, we observed that this region is especially vulnerable to mechanical stress and degenerative changes. The L5-S1 level bears the greatest load of the lumbar spine, making it prone to conditions such as disc degeneration, facet joint osteoarthritis, and ligament thickening, all of which contribute to canal narrowing. In our study, the L5-S1 level had the highest proportion of high-risk stenosis cases, particularly in older females aged 61–75, where 12.2% of patients were classified as high risk. This is consistent with previous findings that suggest the lower lumbar levels, especially L5-S1, are more susceptible to age-related changes, leading to a higher incidence of stenosis. For males aged 61–75, the data indicated that there were 0 borderline-risk cases, 1 high-risk case, and 2 low-risk cases. This highlights the gender differences in stenosis progression at this level, showing that, while they are still at risk, males generally have a lower proportion of high-risk cases compared to females (Table 11).

Table 12 illustrates the average lumbar spinal canal widths measured across different lumbar levels, along with the respective percentages of patients categorized into low-, borderline-, and high-risk groups for stenosis. Additionally, it presents the correlation coefficients that indicate the relationship between age and spinal canal width, showing a consistent negative correlation across all levels. As age increases, the width of the spinal canal tends to decrease, which has significant clinical implications for assessing the risk of lumbar spinal stenosis. The statistical methods used for analysis included independent *t*-tests for gender comparisons and one-way ANOVA to evaluate differences across age groups. The significant *p*-values (*p* < 0.05) suggest that age is a critical factor in determining spinal canal dimensions, further underlining the necessity for age- and gender-specific assessment when evaluating lumbar stenosis risk. This information can serve as a foundation for understanding how anatomical variations in spinal canal width may influence the development of symptomatic stenosis, particularly in older populations.

## 4. Discussion

The present study aimed to assess the risk of lumbar stenosis across various levels of the lumbar spine, with a particular focus being directed to the L4-5 level given its biomechanical significance and the tendency for degenerative changes in this region. The analysis demonstrated that, while most of the patients fell into the low-risk category, the proportion of individuals classified as borderline or high risk increased at lower lumbar levels, particularly at the L4-5 and L5-S1 levels [16]. This finding aligns with the known patterns of age-related spinal degeneration, where the lower lumbar spine is subjected to greater mechanical stress and, consequently, develops narrowing more frequently.

### 4.1. Stenosis Risk Across Lumbar Levels

The overall analysis of stenosis risk across the lumbar spine revealed a consistent trend: the risk of stenosis progressively increased from the upper lumbar levels (L1-2 and L2-3) to the lower levels (L4-5 and L5-S1). At the L1-2 and L2-3 levels, over 87% of patients were classified as low risk, with very few individuals falling into the borderline- or high-risk categories [17]. This is consistent with prior research, which indicates that the upper lumbar levels are less prone to stenosis due to their anatomical configuration and undergo reduced mechanical loading compared to the lower segments.

As expected, the L4-5 and L5-S1 levels showed a notable increase in stenosis risk [18]. At the L4-5 level, 14.63% of patients were classified as borderline, and 7.32% fell into the high-risk category. The L5-S1 level exhibited the highest overall risk, with 12.20% of patients in the high-risk group. These findings highlight the clinical importance of monitoring the lower lumbar levels, particularly in older patients, as these regions are most susceptible to degenerative changes that can lead to symptomatic stenosis.

### 4.2. Gender Differences in Stenosis Risk

When analyzing stenosis risk by gender, a distinct pattern emerged. Females showed a higher proportion of patients in the borderline- and high-risk categories, particularly at the L4-5 and L5-S1 levels. At the L4-5 level, in the 61–75 age group, two female patients were classified as borderline, while one male patient was classified as high risk. Additionally, in the 41–60 age group, one female patient was in the high-risk category, while no males in this age group exhibited high-risk stenosis. These results suggest that females, especially in older age groups, may be more prone to lumbar stenosis at the L4-5 level. The increased stenosis risk in females could be related to several factors, including anatomical differences in pelvic morphology, hormonal changes that affect the musculoskeletal system, and the higher prevalence of osteoporosis, which can contribute to spinal degeneration. Furthermore, females generally have a narrower baseline spinal canal compared to males, which may predispose them to an earlier onset of stenotic changes [19]. In contrast, while males predominantly exhibited low-risk classifications across most age groups, the presence of high-risk cases in the 61–75 age group indicates that they are not without risk. This gender disparity is consistent with previous studies suggesting that males tend to have a wider spinal canal, which may provide a protective effect against stenosis. However, it is important to note that, while males may develop symptomatic stenosis at a later age, the anatomical changes in females seem to manifest earlier, warranting close clinical monitoring in this population [20].

### 4.3. Age-Related Stenosis Risk

Age played a significant role in the distribution of stenosis risk, particularly at the L4-5 level. In the 20–40 age group, the majority of patients were classified as low risk, with only one female patient in the borderline-risk category. This reflects the minimal degenerative changes typically seen in younger individuals. As the patients aged, the proportion of borderline- and high-risk cases increased, especially in females. In the 41–60 age group, one female patient was classified as high risk, indicating that stenosis-related changes are beginning to manifest more clearly in this middle-aged group.

The 61–75 age group, which is generally considered more vulnerable to degenerative spinal changes, showed a higher incidence of stenosis risk, particularly in females. Two females in this group were classified as borderline risk, while one male was classified as high risk, compared to none in the younger male cohort. This pattern suggests that the L4-5 level is a critical site for stenosis development in older adults and that age-related degenerative changes, such as disc herniation, facet joint hypertrophy, and ligamentum flavum thickening, are more pronounced in this region [21,22].

### 4.4. Clinical Implications

The findings of this study have important clinical implications for the early detection and management of lumbar stenosis. Given that the L4-5 and L5-S1 levels are most susceptible to stenosis, routine monitoring of these levels in at-risk populations—particularly older females—could aid in the early identification of spinal canal narrowing before it becomes symptomatic. Early intervention strategies, such as physical therapy, weight management, and lifestyle modifications, could be implemented to slow the progression of degenerative changes and delay the onset of stenosis-related symptoms [23,24].

Additionally, the absence of clinical symptoms in patients with borderline- or high-risk radiographic findings underscores the importance of correlating imaging results with clinical presentation. Not all patients with radiographic evidence of spinal canal narrowing will develop symptomatic stenosis, and careful evaluation of functional limitations, pain levels, and neurological symptoms should guide treatment decisions.

The findings of this study not only highlight the importance of age and gender in assessing lumbar stenosis risk, but they also suggest a future avenue for improving early detection and management using advanced imaging technologies. Given the increasing prevalence of lumbar spinal stenosis in aging populations, the integration of artificial intelligence (AI) algorithms in lumbar MRI scans could offer significant clinical benefits. Automated AI-based tools have the potential to standardize and enhance the detection of spinal canal narrowing by rapidly identifying high-risk patients who may benefit from early intervention, even before clinical symptoms manifest [25,26].

AI models, such as deep learning algorithms, have demonstrated substantial success in medical imaging, particularly in segmentation tasks and abnormality detection. In the context of lumbar spinal stenosis, AI could be used to automatically assess spinal canal dimensions, calculate stenosis risk, and flag patients for further evaluation based on predefined thresholds. This would not only improve the accuracy and consistency of stenosis detection, but it would also reduce the workload for radiologists, allowing them to focus on more complex diagnostic tasks. Moreover, AI-driven analysis could detect subtle changes over time that may be missed during manual assessments, thereby facilitating earlier detection and preventive strategies in at-risk populations, particularly for the L4-5 and L5-S1 levels, which is where degenerative changes are most pronounced.

### 4.5. Comparison with Previous Studies and the Novelty of the Present Work

Focus on Asymptomatic Patients: Unlike many previous studies that have primarily focused on symptomatic patients, this research emphasizes the measurement of lumbar spinal canal dimensions in asymptomatic individuals. This perspective helps to uncover the potential risks and anatomical variations that may not manifest as clinical symptoms [18].

Detailed Age and Gender Analysis: This study offers a detailed analysis of lumbar canal widths across different age groups and genders, providing insights into how these factors influence canal dimensions. This stratified approach enhances the understanding of how demographic variables interact with spinal canal morphology [22].

Comprehensive Measurement Approach: By employing high-resolution MRI imaging to measure spinal canal widths at multiple levels (L1-S1), this work provides a thorough assessment that may inform future diagnostic criteria and clinical practices [12].

Potential Implications for Clinical Practice: The findings may have implications for early diagnosis and management strategies in populations at risk for developing symptomatic stenosis, particularly in asymptomatic individuals who may still experience narrowing of the canal.

### 4.6. Limitations and Future Directions

While this study provides valuable insights into the distribution of stenosis risk across lumbar levels, there are some limitations to consider. First, the study cohort was relatively small, and the findings may not be generalizable to a broader population. Future studies with larger sample sizes and diverse patient populations are needed to validate these results. Additionally, the absence of clinical symptoms in this cohort limits our ability to draw definitive conclusions about the relationship between radiographic stenosis and symptomatic stenosis.

Future research should also explore the impact of comorbidities, such as obesity, osteoporosis, and metabolic diseases, on the progression of lumbar stenosis. Longitudinal studies that track patients over time could provide valuable information on the natural history of spinal stenosis and help identify early markers of disease progression.

Moving forward, we plan to incorporate a more detailed analysis in future studies, where we will conduct the following:

✓ Classify Patients: Divide patients into groups based on spinal canal cross-sectional area measurements, thus allowing us to evaluate those with narrow canals more closely.
✓ Longitudinal Assessment: Utilize MRI data to track changes in spinal canal dimensions over time, providing insights into the progression of stenosis.
✓ Correlation Analysis: Explore the relationship between initial spinal canal dimensions and subsequent narrowing, particularly in older populations.

As lumbar spinal stenosis becomes an increasingly common condition, particularly in older adults, AI-based automated detection systems hold promise for revolutionizing how we assess and manage stenosis risk. Future research should focus on developing and validating AI algorithms that are specifically trained to detect lumbar spinal canal narrowing from MRI images. These models could be designed to measure spinal canal dimensions at multiple levels (L1-2 through L5-S1) and to compare the results against large-scale normative datasets to accurately assess risk.

Furthermore, integrating AI tools with clinical decision support systems could enable clinicians to identify at-risk individuals early, potentially even before they exhibit clinical symptoms. This would facilitate timely interventions such as physical therapy, lifestyle modifications, or even preemptive surgical consultations in more severe cases. AI models could also be trained to recognize patterns of stenosis progression, allowing for longitudinal monitoring of patients and predicting future risk with greater precision.

The implementation of AI in routine clinical practice for lumbar MRI scans could significantly enhance the efficiency and accuracy of spinal stenosis diagnosis, reducing the inter-observer variability commonly seen in manual measurements. In the near future, the combination of advanced imaging technology and AI-based risk prediction could lead to more personalized and proactive care for patients at risk for lumbar spinal stenosis, improving long-term outcomes and quality of life [27].

## 5. Conclusions

This study provides valuable insights into the distribution of lumbar spinal stenosis risk across different lumbar levels, with a particular focus on the L4-5 and L5-S1 regions, where the risk of stenosis is most pronounced. The findings highlight that, while most patients fall into the low-risk category, a significant proportion, especially among older females, exhibited borderline- or high-risk narrowing at the lower lumbar levels. These results underscore the importance of considering both age and gender when assessing the risk of spinal canal narrowing as degenerative changes become more apparent with advancing age, particularly in women.

Despite the presence of radiographic narrowing, none of the patients in this cohort exhibited clinical symptoms of stenosis, reinforcing the need for clinical correlation when evaluating imaging findings. This study also points to the potential of early detection and preventive measures in at-risk populations, with a focus on the close monitoring of the L4-5 and L5-S1 levels, where degenerative changes tend to occur most frequently.

Looking ahead, the integration of artificial intelligence (AI) in lumbar MRI analysis holds great promise for the future of stenosis detection. AI-driven tools could automate the assessment of spinal canal dimensions, offering faster and more precise evaluations that aid in the early identification of stenosis risk before symptoms arise. As lumbar stenosis continues to be a growing concern, particularly in aging populations, innovations in AI technology may pave the way for more personalized and proactive management strategies, ultimately improving patient outcomes and quality of life.

## Figures and Tables

**Table 1:** This table summarizes the average lumbar spinal canal widths (in millimeters) at different lumbar levels (L1-2, L2-3, L3-4, L4-5, and L5-S1) for each age group (20–40, 41–60, and 61–75). The final column shows the average age of the patients in each group.

Age Group	L1-2	L2-3	L3-4	L4-5	L5-S1	AGE
20–40	19.3	19.7	19.0	18.5	18.5	32.8
41–60	19.1	18.8	18.2	18.3	17.9	50.1
61–75	18.5	18.4	16.2	16.0	16.2	66.3

**Table 2:** This table presents the average lumbar spinal canal widths (in millimeters) at each lumbar level (L1-2, L2-3, L3-4, L4-5, and L5-S1) based on gender. The final column shows the average age for male and female patients.

Gender	L1-2	L2-3	L3-4	L4-5	L5-S1	AGE
Female	18.6	18.9	17.6	16.9	16.1	49.3
Male	18.6	18.5	18.3	17.7	17.5	49.4

**Table 3:** This table shows the average spinal canal widths (L1-2, L2-3, L3-4, L4-5, and L5-S1) for each age group and gender, with the average age for each group.

Gender	Age Group	L1-2	L2-3	L3-4	L4-5	L5-S1	AGE
Female	20–40	18.1	19.7	18.7	17.9	16.3	32.8
Female	41–60	18.7	18.8	18.4	17.8	16.6	50.0
Female	61–75	18.5	18.4	16.2	16.0	16.2	66.3
Male	20–40	20.8	21.8	21.3	20.4	21.2	31.0
Male	41–60	19.3	18.9	18.1	18.7	18.6	50.2
Male	61–75	18.4	18.5	16.8	16.5	16.7	66.5

**Table 4:** This table summarizes the results of the *t*-tests comparing genders and ANOVA tests for differences across age groups for each lumbar level.

Lumbar Level	*t*-Test t-Statistic (Gender)	*t*-Test *p*-Value (Gender)	ANOVA F-Statistic (Age)	ANOVA *p*-Value (Age)
L1-2	0.1	0.9	1.5	0.2
L2-3	−0.4	0.7	3.3	0.0
L3-4	0.6	0.5	5.2	0.0
L4-5	0.7	0.5	6.1	0.0
L5-S1	1.3	0.2	3.8	0.0

**Table 5:** Percentage differences in spinal canal widths by age group and gender.

Age Group	Level	Males (mm)	Females (mm)	Percentage Difference (%)	Significance (*p*-Value)
20–40	L1-2	20.8	18.1	14.9	<0.05
	L4-5	20.4	17.9	14.1	<0.05
41–60	L1-2	19.3	18.8	3.0	<0.05
	L4-5	18.8	17.9	5.0	<0.05
61–75	L1-2	18.4	18.5	−0.6	>0.05
	L4-5	16.5	16.1	2.7	>0.05

**Table 6:** The correlation coefficients between the cross-sectional areas of spinal canals and age.

Lumbar Level	Correlation Coefficient (Age vs. Cross-Sectional Area)	*p*-Value	Significance
L1-2	−0.3	<0.05	Significant
L2-3	−0.4	<0.05	Significant
L3-4	−0.4	<0.05	Significant
L4-5	−0.5	<0.05	Significant
L5-S1	−0.4	<0.05	Significant

**Table 7:** The distribution of stenosis risk (low, borderline, and high) across lumbar levels for different age groups.

Age Group	L1-2 Risk	L2-3 Risk	L3-4 Risk	L4-5 Risk	L5-S1 Risk
20–40	Low Risk	Low Risk	Low Risk	Low Risk	Low Risk
41–60	Low Risk	Low Risk	Borderline	Low Risk	Low Risk
61–75	Low Risk	Low Risk	Low Risk	Low Risk	Borderline

**Table 8:** The stenosis risk distribution for males and females at different lumbar levels.

Gender	L1-2 Risk	L2-3 Risk	L3-4 Risk	L4-5 Risk	L5-S1 Risk
Female	Low Risk	Low Risk	Low Risk	Borderline	Low Risk
Male	Low Risk	Low Risk	Borderline	Low Risk	Borderline

**Table 9:** This table shows the percentage of patients classified as low, borderline, or high risk for stenosis at each lumbar level (L1-2, L2-3, L3-4, L4-5, and L5-S1).

Lumbar Level	Low Risk (%)	Borderline Risk (%)	High Risk (%)
L1-2	90.2	9.8	0.0
L2-3	87.8	9.8	2.4
L3-4	82.9	14.6	2.4
L4-5	78.6	14.6	7.3
L5-S1	73.2	14.6	12.2

**Table 10:** L4-5 stenosis risk by age and gender.

Age Group	Gender	Borderline Risk	High Risk	Low Risk
20–40	Female	1	0	5
20–40	Male	0	0	4
41–60	Female	0	1	8
41–60	Male	1	0	6
61–75	Female	2	0	7
61–75	Male	-	-	-

**Table 11:** L5-S1 stenosis risk by age group and gender.

Age Group	Gender	Borderline	High Risk	Low Risk
20–40	Female	0	0	1
20–40	Male	0	0	1
41–60	Female	0	1	0
41–60	Male	0	0	1
61–75	Female	1	0	1
61–75	Male	0	1	2

**Table 12:** Lumbar spinal canal width and stenosis risk.

Lumbar Level	Average Width (mm)	Low Risk (%)	Borderline Risk (%)	High Risk (%)	Correlation Coefficient (Age vs. Width)
L1-2	19.3	90.2	9.8	0.0	−0.3
L2-3	19.7	87.8	9.8	2.4	−0.4
L3-4	19.1	82.9	14.6	2.4	−0.4
L4-5	18.5	78.1	14.6	7.3	−0.5
L5-S1	18.5	73.2	14.6	12.2	−0.4

## Data Availability

The raw data supporting the conclusions of this article will be made available by the author on request.
